# Deciphering protein function during mitosis in PtK cells using RNAi

**DOI:** 10.1186/1471-2121-7-26

**Published:** 2006-06-23

**Authors:** Jane R Stout, Rania S Rizk, Susan L Kline, Claire E Walczak

**Affiliations:** 1 Department of Biochemistry and Molecular Biology, Indiana University Medical Sciences, Bloomington, Indiana 47405, USA; 2 Department of Biology, Indiana University, Bloomington, Indiana 47405, USA; 3 Department of Anatomy and Cell Biology, Indiana University Medical Sciences, Bloomington, Indiana 47405, USA; 4 Ludwig Institute for Cancer Research, University of California San Diego, La Jolla, CA 92093, USA

## Abstract

**Background:**

Studying mitosis requires a system in which the dramatic movements of chromosomes and spindle microtubules can be visualized. PtK cells, due to their flat morphology and their small number of large chromosomes, allow microscopic visualizations to be readily performed.

**Results:**

By performing RNAi in PtK cells, we can explore the function of many proteins important for spindle assembly and chromosome segregation. Although it is difficult to transfect DNA into PtK cells (efficiency ~ 10%), we have transfected a fluorescent siRNA at nearly 100% efficiency. Using a cDNA expression library, we then isolated a complete PtK MCAK (P-MCAK) cDNA. P-MCAK shares 81% identity to Human-MCAK (H-MCAK) protein and 66% identity to H-MCAK DNA. Knockdown of P-MCAK by RNAi caused defects in chromosome congression and defective spindle organization. Live imaging revealed that chromosomes had defects in congression and segregation, similar to what we found after microinjection of inhibitory anti-MCAK antibodies. Because it is laborious to isolate full-length clones, we explored using RT-PCR with degenerate primers to yield cDNA fragments from PtK cells from which to design siRNAs. We isolated a cDNA fragment of the mitotic kinesin Eg5 from PtK cells. This fragment is 93% identical to H-Eg5 protein and 87% identical to H-Eg5 DNA. A conserved 21 bp siRNA was used for RNAi in both HeLa and PtK cells in which Eg5 knockdown resulted in an increased mitotic index and cells with monopolar spindles. In addition, we used RT-PCR to isolate fragments of 5 additional genes, whose sequence identity ranged from 76 to 90% with human, mouse, or rat genes, suggesting that this strategy is feasible to apply to any gene of interest.

**Conclusion:**

This approach will allow us to effectively probe mitotic defects from protein knockdowns by combining genomic information from other organisms with the tractable morphology of PtK cells.

## Background

The mitotic spindle consists of a dynamic array of microtubules and their associated proteins [1,2]. Dynamic microtubules are critical both for spindle assembly and for chromosome movement by capturing chromosomes at the kinetochore, the specialized site where microtubules contact mitotic chromosomes [3,4]. The mitotic spindle is comprised of three classes of microtubules whose dynamics appear to be differentially regulated [5]. Within the spindle, the non-kinetochore or spindle microtubules turn over rapidly; in contrast, the bundles of microtubules in the kinetochore fibers are overall more stable, but locally highly dynamic [6-8]. The astral microtubules are more dynamic relative to interphase microtubules, but it is not known how their stability compares to those of the spindle [9].

Microtubule polymerization dynamics are of fundamental importance for the intracellular functions of the microtubule cytoskeleton and are highly regulated. In general, microtubules in cells turn over much more rapidly than microtubules assembled from pure tubulin *in vitro *[10], due to cellular factors that contribute to increased microtubule turnover, including Op18 [11-15], Tog [16-23], and the microtubule depolymerizing kinesins, MCAK and Kif2A [24-28]. In particular, the essential role that MCAK plays to regulate microtubules in the spindle and at the kinetochore has been the recent focus of much attention.

MCAK is a member of the Kinesin-13 family [29], whose members depolymerize microtubules rather than translocate along them [30-32]. MCAK was originally identified as a protein that localizes to centromeres in mitosis [33], and was shown to be critical for spindle assembly in *Xenopus *egg extracts [24]. MCAK directly destabilizes microtubules by binding to either microtubule end and inducing a conformational change in the microtubule that leads to depolymerization [25,34]. Furthermore, MCAK regulates microtubule dynamics in the cell both during interphase and mitosis [26]. More recently, it has been shown to be a member of the microtubule plus-end tip tracking proteins, but the functional significance of this activity is not known [35,36]. The exact role of MCAK in chromosome movement and segregation has been a subject of debate, specifically, whether it is needed solely for chromosome congression before anaphase or whether it also functions directly in chromosome segregation at anaphase [37-40].

In order to study the exact role of MCAK and other Kinesin-13 family members in the regulation of cellular microtubule dynamics, it is essential to use a cell type in which the dynamics of microtubules during mitosis can be readily visualized. One popular cell type is the marsupial PtK2 cell, from the kidney of a normal adult male *Potorous tridactylis*, which has a large flat morphology and a small number of large chromosomes (for example see [41,42]). However, functional analysis has been limited to the microinjection of inhibitory antibodies or application of small molecule inhibitors due to the lack of genomic information for RNAi knockdown. In addition, these cells generally transfect poorly, which hinders such studies. Here we report the identification of the PtK MCAK gene and the optimization of the techniques to use siRNA-mediated knockdown to deplete endogenous MCAK and compare those effects to those of antibody inhibition. In addition, we use RT-PCR to identify several other partial gene sequences and show the effects of knockdown of additional PtK genes to demonstrate the applicability of our approach.

## Results and discussion

### P-MCAK is homologous to Human and *Xenopus *MCAK

**Figure 1:**
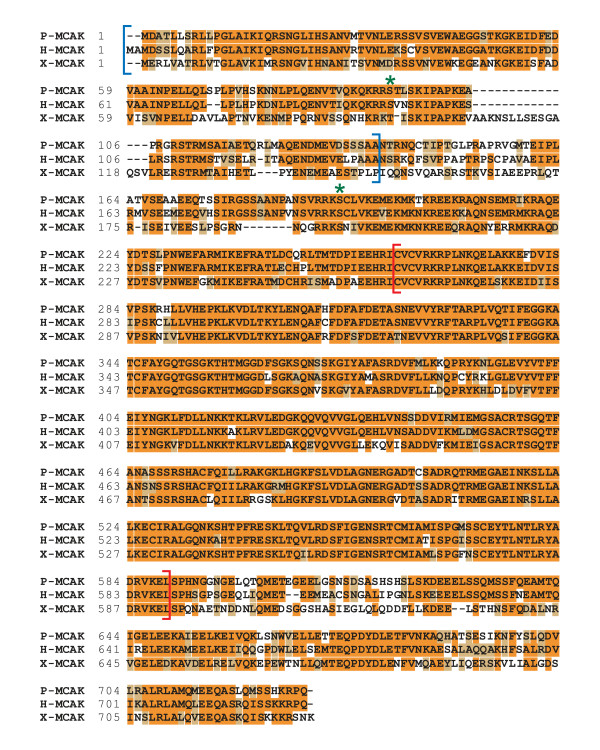
P-MCAK is highly similar to H-MCAK. An alignment of P-MCAK, H-MCAK and X-MCAK proteins is shown. All 3 proteins share significant identity in the N-terminal centromere targeting domain (blue brackets) and the centrally located catalytic core (red brackets). The major Aurora B phosphorylation sites that regulate centromere-targeting and microtubule depolymerization activity are also conserved and are indicated in green.

To isolate a clone encoding P-MCAK, we screened a PtK1 cDNA expression library with an antibody raised against the N-terminus of *Xenopus laevis *MCAK (X-MCAK). We isolated a full-length clone that was 2865 nucleotides in length and coded for a 729 amino acid protein with a predicted MW of 81,552 Da. This protein was 81% identical to Human MCAK (H-MCAK) overall and 92% identical in the catalytic domain (Figure 1). P-MCAK was 66% identical to X-MCAK overall and 86% identical in the catalytic domain. The N-terminal region, which is responsible for targeting MCAK to centromeres, was 76% identical between P-MCAK and H-MCAK and 50% identical between P-MCAK and X-MCAK. Previously identified and functionally important Aurora B phosphorylation sites were also conserved between these three proteins (Figure 1).

### Knockdown of P-MCAK causes defects in spindle morphology and chromosome alignment

**Figure 2:**
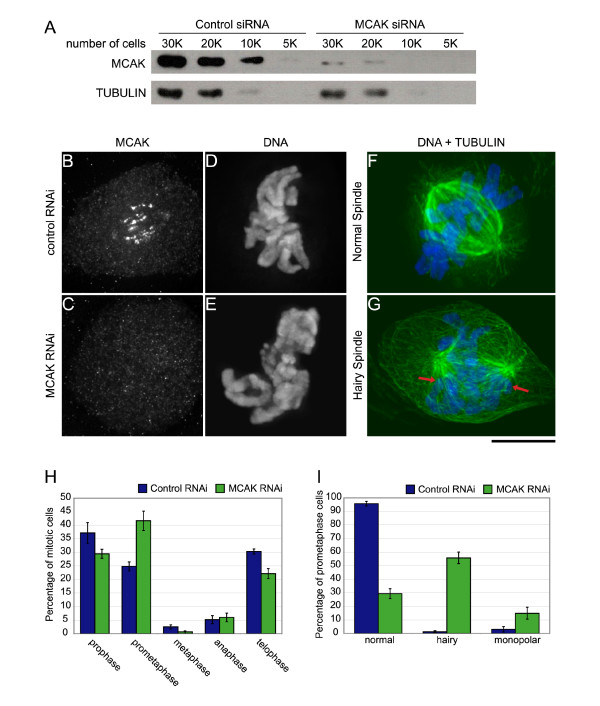
Fixed analysis of P-MCAK knockdown by RNAi shows defects in spindle assembly and a mitotic delay in prometaphase. PtK2 cells were transfected with a 21 bp siRNA specific to P-MCAK and then processed after 72 hrs for immunofluorescence and for immunoblotting. (A) Immunoblot of serial dilutions of either control or MCAK RNAi cell extracts. K indicates the number of cells in thousands loaded in that lane. (B, C, D, E) Immunofluorescence images of control or MCAK RNAi cells show loss of MCAK staining at centromeres. (F-G) Immunofluorescence images of control or MCAK RNAi cells that show an increase in microtubule (green) density during prometaphase as well as an increase in chromosomes (blue) lingering near the poles (arrows) when MCAK levels are knocked down. (H, I) Quantification of mitotic defects caused by MCAK RNAi. At 72 hrs post-transfection, cells were quantified to determine the specificity of defects seen. (H) Knockdown of MCAK causes a prometaphase delay in mitosis. (I) Knockdown of MCAK causes an increase in cells with increased microtubule density. All data represent mean +/- SEM for 6 experiments. N = 600 cells for mitotic index and N = 100 cells for mitotic phenotype counts for each experiment. Scale bar, 10 μm.

To explore the utility of PtK cells for protein knockdown by RNAi, we transfected cells with siGLO, a fluorescently labelled siRNA, and examined them by fluorescence microscopy. We found that nearly 100% of the cells contained the fluorescent dsRNA when examined at 48 hrs post-transfection (not shown). This is in sharp contrast to the small percentage of cells (10–15%) that express a GFP-fusion protein at 48 hrs post-transfection of DNA [26]. To look specifically at the consequences of P-MCAK knockdown, we transfected a nonfluorescent P-MCAK specific 21 bp siRNA into PtK2 cells and examined the cells at 72 hr post-transfection. P-MCAK levels could be reproducibly knocked down by ~96% (range 90–99%) as judged by immunoblot (Figure 2A). In mitotic cells, P-MCAK staining at the centromeres and in the cytoplasm was either no longer visible or greatly reduced (Figure 2B–C). Cells in which P-MCAK levels were knocked down had defects in chromosome alignment and spindle structure (Figure 2D–G), as well as an accumulation of cells in prometaphase (Figure 2H). P-MCAK knockdown cells often had prometaphase chromosome arrangements in which there were numerous chromosomes located at spindle poles (Figure 2G), similar to what has been observed upon expression of a dominant-negative MCAK fragment that targets centromeres in PtK cells [40] and to MCAK RNAi in HeLa cells [43]. A large percentage of bipolar spindles exhibited increased microtubule staining, with excessively long astral microtubules extending toward the cell cortex (Figure 2G, I), which have been referred to as "hairy" spindles [26]. The extent of increased polymer after MCAK inhibition seen in other studies is highly variable with some groups reporting an increase [26,44] (Hedrick and Walczak submitted) and others not seeing a defect in spindle polymer [28,37,43]. Despite these discrepancies, our results are consistent with the idea that one major function of MCAK is to control spindle microtubule dynamics during mitosis to insure proper spindle formation and proper attachment of chromosomes on the spindle.

### Loss of MCAK causes defects in chromosome movement

Previous studies with injection of a centromere dominant-negative form of MCAK [40] or expression of motorless MCAK [37] resulted in an increased number of lagging chromosomes, which are chromosomes that remain in the midzone during anaphase and often into telophase. This is in contrast to our current studies in which knockdown of MCAK by RNAi did not result in lagging chromosomes during anaphase upon analysis of fixed samples (data not shown). This might be because the low frequency of anaphase cells in fixed samples in both control and MCAK RNAi cells hindered our analysis of this defect. Because lagging chromosomes are often caught at the midzone beyond anaphase, we also scored telophase cells for lagging chromosomes. Though our results did show a decrease in the percentage of telophase cells after MCAK RNAi (Fig. 2H), we still did not see an increase in lagging chromosomes (data not shown). Another possibility is that during early anaphase, some chromosomes lag behind the mass of segregating chromosomes, but then recover quickly enough so that these "stragglers" [45] eventually get partitioned to the two daughter cells. This phenotype would be difficult to discern in fixed analysis. To test this possibility, we imaged control and MCAK RNAi cells by time-lapse phase contrast microscopy.

**Figure 3:**
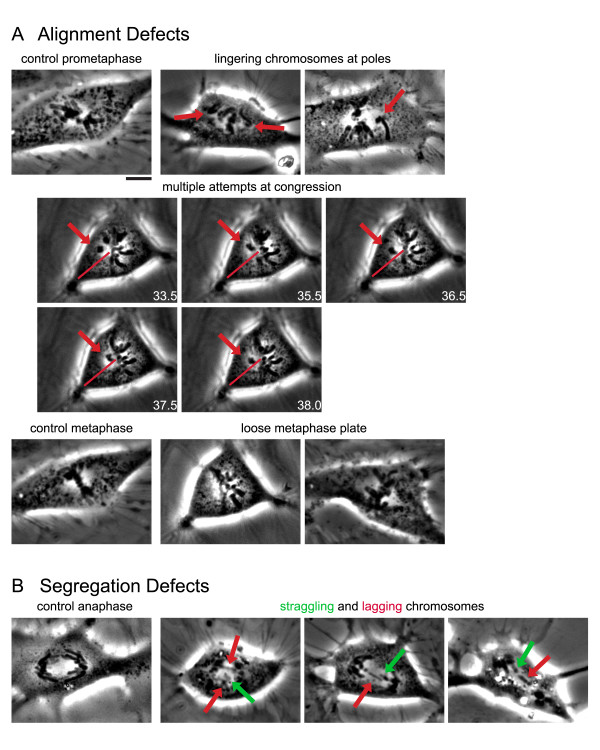
The congression and segregation defects scored in MCAK inhibited cells are displayed. Live imaging of RNAi knockdown and antibody inhibition of P-MCAK show an increase in congression defects, and an increase in segregation defects compared to control RNAi knockdown and IgG injected cells. (A, B) Series of panels from time-lapse phase contrast microscopy of different movies of either control or MCAK inhibited cells that illustrate the different mitotic defects that were scored in all cells. Images chosen are to represent a particular morphological defect and include images taken from both antibody injection experiments and RNAi experiments. (A) Alignment defects and (B) segregation defects. In each panel, the arrow points to the chromosome of interest, and the line indicates the trajectory of chromosome movement and can serve as a reference point for the extent of chromosome movement. Scale bar, 10 μm.

Time-lapse movies were scored for congression and segregation defects (Figure 3A, B, Table 1 and Videos 1 and 2). The most common defects were in chromosome congression. There was an increase in the percentage of cells that had chromosomes lingering near the poles for extended periods of time compared to control cells. There was also an increase in the percentage of cells that underwent multiple attempts at congression, which was defined as any chromosome that regressed toward the pole after it had congressed more than half the distance from the spindle pole to the metaphase plate (Figure 3A, Video 2). In addition, there was an increase in the percentage of cells in which not all chromosomes congressed to the metaphase plate prior to the onset of anaphase. The most striking defect was that in MCAK RNAi cells, nearly all cells had a dispersed, or "loose", metaphase plate, which coincided with chromosomes that exhibited high oscillations (Figure 3A), similar to what we observed previously after injection of a centromere dominant-negative MCAK [40]. Despite these defects, there was no significant alteration in the timing of mitosis from nuclear envelope breakdown until anaphase onset. As for lagging chromosomes, we found only a small percentage of cells with chromosomes remaining at the spindle equator after segregation; we often found that these laggers appeared to be corrected late in mitosis when they would quickly move toward one pole or the other [45]. However, there was a higher incidence of "straggling" chromosomes, which trailed behind the segregating chromosomal mass (Figure 3B). The appearance of straggling chromosomes correlated with high oscillations at the metaphase plate. This may be because, in a highly oscillating pair, the sister chromatid most distal to the pole to which it will eventually segregate could appear as a straggler because it started anaphase away from the main chromosomal mass. In the phase movies that we observed it is difficult to definitively track individual chromosomes through the chromosomal mass as they separate to their respective poles.

**Table 1:** Analysis of the morphological defects and mitotic progression of mitosis in live images of RNAi transfected cells vs. antibody-injected cells.

**Measurement**	Control RNAi	MCAK RNAi	Control IgG	Anti-MCAK
**Alignment Defects**	% cells (n)	% cells (n)	% cells (n)	% cells (n)
Chromosomes lingering at poles	26 (17)	51 (15)	18 (17)	38 (16)
Multiple attempts to congress	33 (17)	49 (15)	18 (17)	56 (16)
Not all chromosomes congress	4 (16)	13 (13)	6 (17)	27 (16)
Loose alignment with high oscillations	13 (16)	89 (16)	24 (17)	87 (16)
Total % cells with alignment defects	39 (18)	88 (16)	41 (17)	88 (17)
**Segregation Defects**	% cells (n)	% cells (n)	% cells (n)	% cells (n)
Straggling chromosomes	11 (17)	53 (12)	25 (16)	50 (16)
Lagging chromosomes	0 (17)	6 (12)	6 (16)	23 (13)
Total % cells with segregation defects	12 (17)	42 (12)	25 (16)	50 (16)

**Measurement**	Control RNAi	MCAK RNAi	Control IgG	Anti-MCAK

Spindle Defects	Avg. ± SEM (n)	Avg. ± SEM (n)	Avg. ± SEM (n)	Avg. ± SEM (n)
Spindle length (μm)	15.3 ± 0.5 (14)	12.9 ± 0.5* (12)	14.5 ± 0.6 (16)	12.9 ± 0.4* (17)

**Timing of Mitotic Progression**	Avg. ± SEM (n)	Avg. ± SEM (n)	Avg. ± SEM (n)	Avg. ± SEM (n)
Time between injection and NEB (min)	NA	NA	5.6 ± 2.2 (17)	2.8 ± 0.8 (18)
Time between NEB and LCC (min)	17.4 ± 1.3 (14)	23.3 ± 7.0 (12)	16.1 ± 1.7 (15)	32.3 ± 3.4* (17)
Time between LCC and Ana A (min)	13.7 ± 2.7 (13)	12.6 ± 1.8 (11)	16.1 ± 1.7 (14)	17.4 ± 2.2 (18)
Total time between NEB and Ana A (min)	30.1 ± 2.7 (17)	32.6 ± 5.9 (14)	33.3 ± 1.7 (17)	47.7 ± 4.1* (18)

NEB, Nuclear envelope breakdown, LCC, the time when the last chromosome begins congressing. NA, not applicable. * Represents a p-value of <0.05 and indicates a significant difference in spindle length or timing.

In addition to chromosome congression and segregation defects, we determined that MCAK RNAi cells also displayed an overall shorter spindle length (Table 1). This result may at first seem counterintuitive. Because we have shown that depletion of MCAK results in increased astral microtubule length, it could be expected that its depletion would also result in longer spindle microtubules increasing the overall spindle length. However, the increased length of the astral microtubules seen upon the depletion of MCAK may instead sufficiently reduce the overall tubulin pool available so that normal spindle length cannot be maintained in the dividing cell. Alternatively, it has been shown previously that treatment of cells with low concentrations of paclitaxel, which suppress microtubule dynamics without causing a significant change in microtubule polymer, also result in shorter spindles [46]. The authors interpreted this data to indicate that paclitaxel suppressed plus-end dynamics of kinetochore microtubules without affecting the depolymerization at poles due to flux. Inhibition of MCAK may similarly be suppressing plus-end dynamics of kinetochore microtubules. Consistent with this idea, we reported previously that injection of the MCAK centromere dominant-negative also caused shorter spindles [5].

To determine if the defects in MCAK RNAi cells were similar to those we have described earlier with fixed analysis of cells after antibody microinjection, we imaged cells by time-lapse phase contrast microscopy after antibody injection [26]. Prophase cells were injected with either control IgG or anti-MCAK antibodies and then followed through mitosis by phase-contrast microscopy. Similar to the MCAK RNAi cells, the MCAK antibody injected cells had defects during chromosome congression (Figure 3, Table 1, and Videos 3 and 4). Chromosomes lingered at spindle poles, had difficulty in congression and most significantly had increased oscillations at the metaphase plate, which resulted in a loose metaphase plate and perhaps the higher incidence of straggling chromosomes at anaphase. Similar to MCAK RNAi cells, anti-MCAK antibody injected cells also had shorter spindles. One difference between antibody-injected and RNAi cells is that although MCAK RNAi caused a slight increase in lagging chromosomes, the antibody-injected cells had a higher incidence of lagging chromosomes. However, the overall percentage of cells with one or more segregation defects was only slightly higher in MCAK antibody injected cells than in MCAK RNAi cells.

It is not clear why the segregation defects translate to fewer lagging chromosomes in MCAK RNAi knockdown cells versus MCAK antibody inhibited cells. One possibility is that cell-to-cell variability with RNAi knockdown and the lower numbers of cells in our live analysis contributed to the lower overall percentage of lagging chromosomes in MCAK RNAi cells. Unfortunately we could not fix and stain live cells imaged after MCAK RNAi to determine the extent of knockdown in the imaged cell because MCAK staining is quite diffuse in the cytoplasm and in the nucleus at early G1. However, the failure to see lagging chromosomes in fixed analysis of both anaphase and telophase cells where we had analyzed many more cells suggests that these results are not simply due to inefficient knockdown in the imaged cells. It could be that antibody inhibition blocks MCAK activity in ways that RNAi does not. Antibody inhibition may impede MCAK function locally in the cell, such as at the centromere, and this contributes to the increase in lagging chromosomes in ways that global knockdown by RNAi would not, or it could be that our antibodies are interfering with the function of another protein that possibly interacts with MCAK producing a cumulative effect. We think both of these possibilities are unlikely because the antibody-antigen complexes are found in the cytoplasm, not at the centromere [26], and because we have injected 3 different antibodies, including a newly developed one to the P-MCAK protein, as well as a dominant-negative version of MCAK, and all give similar phenotypes. Another possibility is that RNAi knockdown depletes the protein over a longer time period than antibody injection, which may allow a compensatory mechanism to be activated. In the antibody injection studies, antibodies are injected in a short window of time prior to nuclear envelope breakdown at a time when the microtubules are extremely dynamic, so the effects of the MCAK antibody inhibition are immediate and dramatic, and the cell has no time to invoke any compensatory mechanism. In support of this idea, during the optimization of our knockdown conditions in which the knockdown of MCAK was less efficient (~70%), we did see more lagging chromosomes at anaphase in our fixed analysis (data not shown). It could be that the lower percentage knockdown of MCAK in these initial transfections did not initiate a second compensatory mechanism and therefore more closely resembled the defects associated with MCAK antibody inhibition.

We also measured the timing of mitotic progression in both the RNAi cells and in the antibody-injected cells (Table 1). Previously we had shown that injection of the dominant-negative MCAK, GFP-CEN, causes a delay in prometaphase [40]. The knockdown of MCAK by RNAi in our live analysis did not result in a significant increase in the time between nuclear envelope breakdown and anaphase A onset, however MCAK antibody microinjection did cause a prometaphase delay. These differences may also be explained by the timing of the experiments in that the antibodies are injected just prior to nuclear envelope breakdown. In addition, it is important to note that while the live analysis did not show a major defect in timing, there were several individual live imaged cells that did show a prometaphase delay, and the fixed analysis of cells clearly showed an increase in the percentage of cells in prometaphase, suggesting that with a larger population of cells, such as our fixed analysis, the delay can be detected.

Overall, from our analysis of MCAK RNAi knockdown phenotypes, we conclude that RNAi in PtK2 cells is an effective tool to study the roles of mitotic proteins. Our results with P-MCAK RNAi corroborate previous work showing loss of MCAK leads to aberrant spindle formation as well as aberrant kinetochore-microtubule attachments that lead to improper chromosome congression and missegregation of chromosomes during anaphase. From these studies we also conclude that RNAi and antibody injection studies do give similar overall results, but more subtle details may vary between the methods because of the dramatic difference in the time-course of the inhibition. We feel that these two approaches should therefore be considered complementary rather than strictly redundant.

### Partial cDNA sequences can be used to develop SiRNAs

**Figure 4:**
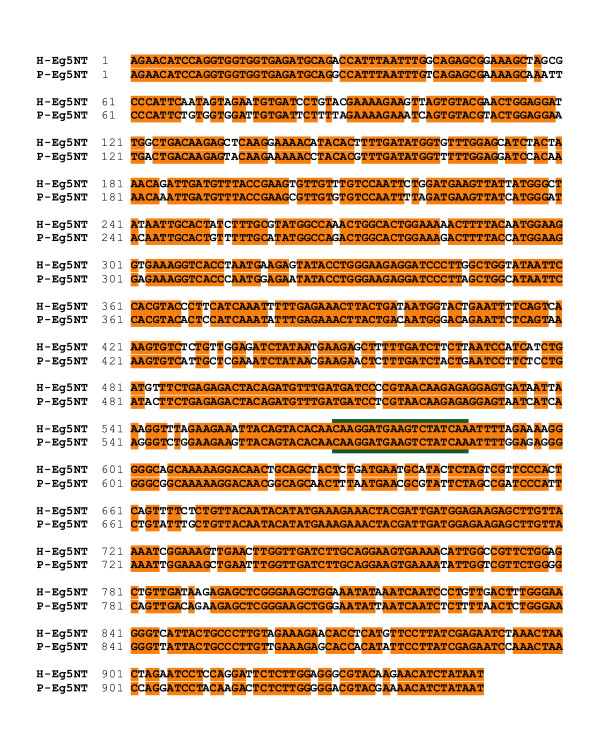
P-Eg5 and H-Eg5 share significant identity in their catalytic domains. A series of degenerate oligonucleotides were used to clone a segment of both the H-Eg5 and P-Eg5 cDNA by RT-PCR. (A) An alignment of P-Eg5 and H-Eg5 DNA is shown. The sequence chosen for the 21 bp siRNA is outlined in green.

**Figure 5:**
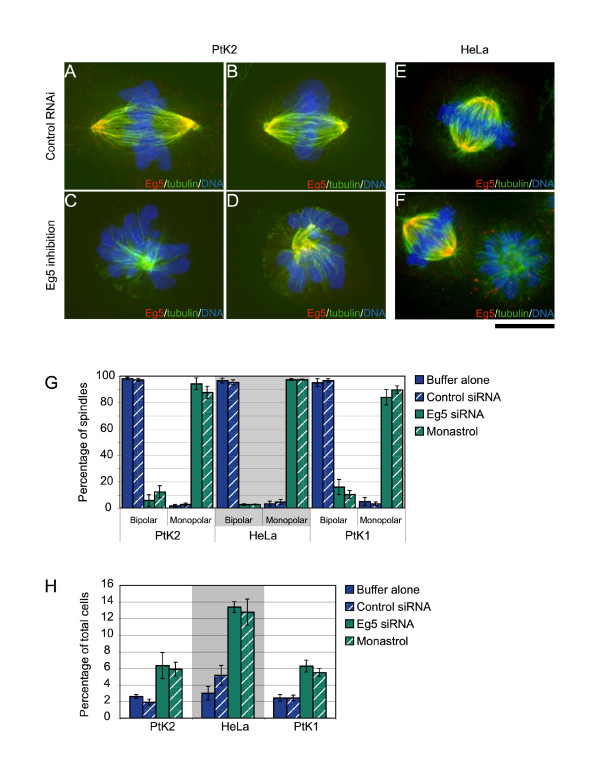
Knockdown of Eg5 causes an increase in monopolar spindles. Either PtK2 (A, B, C, D) or HeLa (E, F) cells were transfected with the identical 21 bp siRNA to knockdown Eg5 levels. (A, B, E) Control cells stained to visualize microtubules (green), Eg5 (red) and DNA (blue). Eg5 knockdown cells (C, F) or cells that were treated with the Eg5 inhibitor monastrol (D) were stained to visualize microtubules (green), Eg5 (red) and DNA (blue). Knockdown of Eg5 levels resulted in cells with monopolar spindles and a mitotic delay as reported previously. (G) Quantification of the percentage of RNAi cells with bipolar versus monopolar spindles in either buffer, negative control siRNA, Eg5 siRNA, or monastrol treated cells for PtK2, HeLa and PtK1 cell lines. (H) Quantification of the mitotic index in PtK2, HeLa, and PtK1 cells transfected with buffer, control siRNA, or Eg5 siRNA, or treated with monastrol. All data represent mean +/- SEM for 3+ experiments. N = 600 cells for mitotic index and N = 100 cells for mitotic phenotype counts for each experiment. Scale bar, 10 μm.

Because it is laborious to clone out full-length cDNAs, we tested whether we could use RT-PCR to obtain a partial cDNA sequence. We chose Eg5 as our test candidate because it is present across a wide phylogeny of organisms and its inhibition by antibody injection, immunodepletion, RNAi, small molecule inhibitors or genetic knockouts results in monopolar spindles in nearly every organism [47-52]. Using RT-PCR, we were able to clone a portion of the PtK Eg5 (P-Eg5) catalytic domain that was 93% identical to H-Eg5 protein and 87% identical to H-Eg5 DNA (Figure 4). Utilizing this information, we generated a 21 bp siRNA that was identical between P-Eg5 and H-Eg5 (Figure 4) and used it for knockdown of Eg5 in both PtK2 cells and HeLa cells. Whereas spindles in control cells were predominantly bipolar (Figure 5A, B, E), knockdown of Eg5 in PtK2 cells (Figure 5C) or HeLa cells (Figure 5F) caused an increase in cells with monopolar spindles, similar to cells treated with monastrol, the small molecule inhibitor of Eg5 (Figure 5D). Although we were not able to determine the level of knockdown by immunoblotting because no antibodies gave sufficiently strong signals on western blots of PtK2 cells, it was clear that any bipolar spindles that formed still contained substantial Eg5 staining, whereas all monopolar spindles had no or extremely little residual Eg5 staining (Figure 5F). Thus the percentage of monopolar structures formed after Eg5 knockdown is likely a good indicator of the effectiveness of the knockdown. We found that in PtK2 cells, nearly 97% of control cells had bipolar spindles whereas >94% of the structures formed in the absence of Eg5 were monopolar compared to ~88% monopolar spindles when treated with the Eg5 small molecule inhibitor (Figure 5G). Similar to what was observed in PtK2 cells, inhibition of Eg5 in HeLa cells also caused a dramatic increase in the percentage of cells with monopolar spindles, ~97% with both Eg5 RNAi and monastrol (Figure 5G). Both cell types show an ~3-fold increase in the mitotic index after knockdown of Eg5 (Figure 5H), indicating that knockdown of Eg5 induces a mitotic block in both cell types. HeLa cells treated with control siRNA have a higher mitotic index than PtK2 cells treated with control siRNA, so it is not necessarily surprising that the percentage increase of cells blocked in mitosis is higher in HeLas. Together these results show that the phenotypic consequence of Eg5 knockdown in PtK2 cells is comparable to that obtained in HeLa cells or by utilization of a small molecule inhibitor.

In order to determine that efficient RNAi in PtK cells is not limited to our PtK2 cell line, we repeated the Eg5 RNAi knockdown in PtK1 cells, which are from the kidney of a normal adult female *Potorous tridactylis*. Like PtK2 cells, inhibition of Eg5 by either RNAi or monastrol treatment also caused monopolar spindles with no or residual Eg5 staining in RNAi cells (data not shown). Nearly 97% of the control cells had bipolar spindles compared to 84% monopolar spindles in Eg5 RNAi PtK1 cells or the ~90% in monastrol treated cells (Figure 5G). The mitotic index also increased by more than 2-fold just as in PtK2 cells. These results along with those above show that RNAi in PtK cells is a viable method to study the function of mitotic proteins in these morphologically advantageous cells.

### Identification of additional PtK2 partial clones

To demonstrate that our approach using RT-PCR would allow us to readily identify homologous sequences from PtK cells, we chose 4 other genes from which to obtain partial sequences as well as one other gene in which we had a full-length cDNA (Tables 2 and 3). In each case, we were able to obtain a cDNA sequence of ~350–2200 bp that ranged in identity from 76–90% with human, mouse or rat sequences (Table 2). There was no organism that consistently had greater identity with the PtK sequences than other organisms, suggesting that it is not advantageous to rely on one sequenced genome over another for design of degenerate oligonucleotide primers. We next investigated if it is routinely possible to utilize genomic sequence from human, mouse or rat to generate mixtures of siRNAs using for example RNAse III digestion of dsRNA [53]. We analyzed how many 21 bp stretches were conserved between PtKs and other organisms (Table 2). Overall this number was very low ranging from 1 21 bp stretch out of a 2166 bp gene to a high of 27 21 bp stretches out of a 2040 bp gene. Most commonly the matches were in the range of 1–3 21 bp stretches. These data show that the most efficient approach is to use RT-PCR to isolate a small stretch of sequence of the PtK gene and then use this sequence to design siRNAs.

**Table 2:** Homologous genes across mammalian species share a high degree of identity at the DNA level.

	Percent identity between paired alignments			# identical 21 base pair stretches in paired alignments			# nucleotides compared			
	
	P-H	P-M	P-R	P-H	P-M	P-R	P	H	M	R
	
MCAK FL	66.4	62.1	58.6	3	1	2	2187	2178	2166	2016
Eg5 (18–346)	86.6	85.0	84.1	8	4	5	990	990	990	990
Kif2a FL	88.9	80.9	75.6	27	18	15	2040	2040	2151	2316
KifC1 (518–768)	78.4	78.8	78.2	1	2	1	760	754	754	760
Kif18a (110–383)	83.1	80.0	79.7	3	2	2	824	824	824	425
NFYB (64–200)	86.5	83.5	82.3	2	0	0	413	413	413	413
RPLP0 (49–165)	89.8	88.1	87.0	4	1	2	353	353	353	353

P, *Potorous tridactylus *sequence; H, *Homo sapiens *sequence; M, *Mus musculus *sequence; R, *Rattus norvegicus *sequence. MCAK and Kif2a are kinesin-13 family members; Eg5 is a kinesin-5 family member; KifC1 (HSET) is a kinesin-14 family member; Kif18a is a kinesin-8 family member; NFYB is a histone-like protein analogous to H2B; RPLP0 is ribosomal protein, large, PO. FL, full-length protein. Accession numbers can be found in Table 3.

**Table 3:** Accession Numbers for DNA sequences used to compare to PtK2 RTPCR fragments

	Human	Mouse	Rat
MCAK	NM_006845	NM_134471	NM_134472
Eg5	NM_004523	NM_010615	XM_215287
Kif2a	NM_004520	NM_008442	XM_345150.1
KifC1	XM_371813.2	NM_016761.1	NM_001005878.1
Kif18a	NM_031217	NM_139303.1	XM_342486.1
NFYB	NM_006166.3	NM_010914.1	NM_031553.2
RPLP0	NM_001002	BC011106.1	NM_022402

## Conclusion

Here we have developed a method to use genomic information from other organisms with a sequenced genome to knockdown protein levels in PtK cells. This will allow us to systematically explore the roles of mitotic proteins taking advantage of the excellent morphology and ease with which live analysis of cellular dynamics can be performed in PtK cells. While our interest is in understanding mitosis and PtK cells provide a model system to research this process, the approach we outlined here should be adaptable to any cell type regardless of the existence of a sequenced genome. In addition, our data showing that the efficiency of transfection between DNA and siRNAs can be significantly different suggests that other cell types that have been experimentally intractable to transfection techniques may be able to be utilized for gene silencing by siRNAs.

## Methods

### Cloning of P-MCAK and P-Eg5

A cDNA expression screen of a PtK1 cDNA library was carried out utilizing conventional methods [54]. An antibody raised against the N-terminus of X-MCAK [24] was used as a probe in the screen. We screened 900,000 plaques and isolated a single full-length clone encoding P-MCAK [GenBank: DQ444242]. To clone a portion of the catalytic domain of P-Eg5, we generated degenerate oligonucleotides by aligning human, mouse, and rat sequences encoding Eg5 and used these in RT-PCR reactions (Qiagen one-step RT PCR kit) using total RNA isolated from PtK2 cells (Qiagen RNAeasy) as a template. The Eg5 fragments were then subcloned into pEGFPC1 (Clontech) plasmid and fully sequenced on both strands. Sequence analysis was performed using Sequencher (Gene Codes Corporation), and the percent identity of fragments was calculated using DNA Strider.

### SiRNA transfections

PtK2 cells were plated at 15,000 – 20,000 cells/ml, and PtK1 cells were plated at 20,000 – 25,000 cells/ml in D-MEM (Invitrogen) supplemented with penicillin/streptomycin and 10% FBS 24 hrs before transfection onto coverslips in 6-well dishes and grown at 37°C, 5% CO_2_. Cells were then transfected with a non-fluorescently labelled 21 bp siRNA (Dharmacon) to P-MCAK (UGACUUUCUUUGAGAUCUA [dT] [dT]), or Eg5 (NNCAAGGAUGAAGUCUAUCAA), and either control oligo GFP (GCAAGCUGACCCUGAAGUUCAU) or siCONTROL Non-Targeting siRNA #2 to luciferase using Oligofectamine (Invitrogen) following their protocol with the following exceptions: 200 pmol siRNA/35 mm well was used, siRNA/lipid complexes were formed in D-MEM without penicillin/streptomycin and FBS, complexes were diluted in D-MEM/10% FBS, and 1 ml of siRNA/lipid dilution was added to each well the day of transfection, with an additional 1 ml/well of D-MEM/10% FBS added 24 hr post-transfection. Cells were examined at 48 hrs for Eg5 and 72 hrs for MCAK post-transfection. These time points were chosen after a qualitative analysis of the efficiency of knockdown by phenotypic analysis of spindle morphology. To determine the efficiency of transfection, cells were plated identically as above except that they were transfected with siGlo Risc-free RNA (Dharmacon) and then analyzed at 24–72 hrs post-transfection. No differences in the efficiency of transfection were observed by determining the percentage of cells containing fluorescent RNA. It should be noted that during our optimizations, we found that silencing efficiency decreased with increased number of passages beyond 6–8 weeks after thawing the cells. For HeLa cell transfections of Eg5, cells were plated at 5,000 – 6,000 cells/ml 24 hrs before transfection and then processed identically as described for PtK cells except that cells were passaged and transfected in Opti-MEM (Invitrogen) and processed 24 hrs post transfection. For treatment with monastrol, both HeLa and PtK cells were plated at the same density as for RNAi, grown for either 24 hrs for HeLa cells or 48 hrs for PtK cells, at which time the growth media was replaced with media containing 100 μM monastrol, and the cells incubated for a further 24 hrs. Monastrol treated cells were fixed and stained identically to the Eg5 siRNA transfected cells.

### Immunofluorescence

For Eg5 staining, HeLa cells or PtK cells were rinsed in PBS (12 mM Phosphate, 137 mM NaCl, 3 mM KCl, pH 7.4) and then fixed for 5' in -20°C MeOH. All subsequent immunofluorescence-labelling procedures were performed at room temperature. Fixed cells were rinsed with TBS-TX [TBS (20 mM Tris, 150 mM NaCl, pH 7.5) + 0.1% Triton X-100] and blocked in AbDil (2% BSA, 0.1% NaN_3 _in TBS-TX) for 30 min. All subsequent rinses between antibody incubations were performed using TBS-TX, and all antibodies were diluted in AbDil. Anti-H-Eg5 antibody (1/500 dilution; Cytoskeleton) was incubated for 30' on cells followed by incubation in 1/50 fluorescein-conjugated goat anti-rabbit secondary antibody (Jackson Immunologicals). For staining of microtubules, cells were incubated for 30 min in DM1alpha (Sigma), 1/500 for PtK cells and 1/1000 for HeLa cells, followed by a 30 min incubation in 1/50 Texas Red-conjugated donkey anti-mouse secondary antibody (Jackson Immunological). DNA was visualized using 2 μg/ml Hoechst in TBS-TX for 5 min. Coverslips were mounted onto microscope slides using 0.5% p-phenylenediamine, 20 mM Tris (pH 8.8) in 90% glycerol. For immunofluorescence of MCAK, cells were fixed for 20 min in PHEM (60 mM Pipes, 25 mM Hepes, 10 mM EGTA, 4 mM MgSO_4_, pH 7.0) supplemented with 2% formaldehyde. Cells were blocked in AbDil for 30' and then incubated for 30 min in 5 μg/ml anti-XMCAK-CT [25] followed by a 30 min incubation in 1/50 fluorescein-conjugated goat anti-rabbit secondary antibody (Jackson Immunologicals). For microtubule staining after MCAK knockdown, cells were fixed and processed as described for MCAK staining except that the fix was supplemented with 4% formaldehyde and 0.1% glutaraldehyde. Free aldehydes were quenched in TBS plus trace NaBH_4 _for 5 min.

### Microinjections and microscopy

Microinjections were performed essentially as described previously [26]. Time-lapse analysis was performed 70–87 hrs post-transfection using a Nikon TE-300 inverted microscope and a 40x/0.60 numerical aperture Plan Fluor objective (Nikon). Specimens were maintained at 36–37.5°C by using a 400 ASI airstream incubator (Nevtek). Phase contrast images were collected with a Cool Snap Cf camera (Roper Scientific) at 30-s intervals at 100-ms exposure times, and all cameras, shutters, and filter wheels were controlled by MetaMorph. Micrographs were assembled in Adobe Photoshop for identical contrast enhancement, and montages were prepared using Adobe Illustrator. Movies were prepared in MetaMorph and converted to Quicktime format.

Fixed cells were imaged on a Nikon E-600 microscope equipped with 40X/1.0 NA Plan Apo oil, 60X/1.4 NA Plan Apo oil, and 100X/1.3 NA Plan Fluor oil objectives (Nikon). Digital images were collected with either a Micromax 1300 Y cooled CCD camera or a CoolSnap HQ (Roper Scientific, Inc.). All cameras, shutters, and filter wheels were controlled by MetaMorph software (Molecular Devices). Z-series optical sections through each cell were obtained at 0.5 μm steps with the use of MetaMorph software and a stepping motor (Prior Scientific Instrument). Images were then deconvolved (blind iterative deconvolution) using AutoDeblur 9.3 software (AutoQuant Imaging). All micrographs were assembled in Adobe Photoshop CS for contrast enhancement, and montages were prepared using Adobe Illustrator CS.

### Immunoblotting

For determining the amount of protein knockdown by immunoblotting, PtK2 cells were harvested by trypsinization at 72 hrs post-transfection, washed in PBS and then counted. Equal numbers of cells (serially diluted) were run on SDS-PAGE followed by immunoblotting. The blots were simultaneously probed with anti-XMCAK NT2 at 2 μg/ml in AbDil-T (made with 0.1% Tween-20 instead of TX-100) and DM1alpha (1/5000), washed in TBST, followed by incubation in a mixture of 1/20000 of anti-rabbit and 1/20000 anti-mouse horse-radish peroxidase secondary antibodies. The blots were washed and developed using Pierce SuperSignal WestPico ECL reagents. Scanned images of the western were quantified using ImageJ 1.34s v10.2 (NIH).

### Quantification and analysis

Mitotic indices were calculated for both control and experimental conditions by scoring mitotic versus interphase cells for 200–300 cells/per coverslip for a total of 600 cells and taking the percentage of mitotic cells per cell total. To calculate the monopolar phenotype of Eg5, 100 mitotic cells between prometaphase and anaphase (when there is normally a bipolar spindle in control cells) were scored as either bipolar or monopolar spindles, and the percentages for both control and experimental cells were calculated. For MCAK RNAi analysis, 100 mitotic cells of both control and MCAK RNAi cells were scored for mitotic cell stage, and for monopolar spindles, for "hairy" (excess microtubules) prometaphase cells, and for lagging chromosomes in anaphase or telophase. The mean and SEM for 3 or more experiments are reported. Significance was considered if p < 0.5 as calculated by a paired t-test using F-test two-sample to determine variance. For analysis of time-lapse movies, the movies were coded and then scored blind by two individuals for presence or absence of the described mitotic defects. Any discrepancies were then scored blind by a third individual. All scores are reported as the percentage of cells scored for each defect. Distance measurements of spindle length were made using MetaMorph software. The spindle length was measured from the time-lapse movie at the frame of anaphase A onset. Measurements were collected in pixel number then converted to micrometers. All calculations for fixed and live analyses were performed in Excel.

## Authors' contributions

JRS performed the majority of the experiments in the paper, assembled all figures and assisted in writing of the manuscript. RSR performed the antibody microinjection experiments, the phenotypic analysis and the timing analysis of the movies. SLK developed the antibody injection protocol and image analysis and did the phenotypic analysis of the time-lapse movies as well as consulted on the design and quantification of many experiments. CEW performed some of the experiments, assisted in the phenotypic analysis and oversaw the design and implementation of all of the experiments as well as the writing of the manuscript.

## Supplementary Material

Additional File 1 Movie 1 – Control siRNA transfected cell Click here for file

Additional File 2 Movie 2 – P-MCAK siRNA transfected cell Click here for file

Additional File 3 Movie 3 – IgG injected cell Click here for file

Additional File 4 Movie 4 – anti-MCAK injected cell Click here for file

## References

[B1] Scholey JM, Brust-Mascher I, Mogilner A (2003). Cell division. Nature.

[B2] Wittmann T, Hyman A, Desai A (2001). The spindle: a dynamic assembly of microtubules and motors. Nat Cell Biol.

[B3] Biggins S, Walczak CE (2003). Captivating capture: how microtubules attach to kinetochores. Curr Biol.

[B4] Cleveland DW, Mao Y, Sullivan KF (2003). Centromeres and kinetochores: from epigenetics to mitotic checkpoint signaling. Cell.

[B5] Kline-Smith SL, Walczak CE (2004). Mitotic spindle assembly and chromosome segregation: Refocusing on microtubule dynamics. Mol Cell.

[B6] Gorbsky GJ, Borisy GG (1989). Microtubules of the kinetochore fiber turn over in metaphase but not in anaphase. J Cell Biol.

[B7] Mitchison TJ, Evans L, Schultze E, Kirschner MW (1986). Sites of microtubule assembly and disassembly in the mitotic spindle. Cell.

[B8] Mitchison TJ, Salmon ED (1992). Poleward kinetochore fiber movement occurs during both metaphase and anaphase-A in newt lung cell mitosis. J Cell Biol.

[B9] Rusan NM, Fagerstrom CJ, Yvon AM, Wadsworth P (2001). Cell cycle-dependent changes in microtubule dynamics in living cells expressing green fluorescent protein-alpha tubulin. Mol Biol Cell.

[B10] Desai A, Mitchison TJ (1997). Microtubule polymerization dynamics. Annu Rev Cell Dev Biol.

[B11] Belmont L, Deacon HW, Mitchison TJ (1996). Catastrophic revelations about Op18/stathmin. Trends Biochem Sci.

[B12] Marklund U, Larsson N, Melander H, Gullberg M (1996). Oncoprotein 18 is a phosphorylation-responsive regulator of microtubule dynamics. EMBO J.

[B13] Larsson N, Marklund U, Gradin HM, Brattsand G, Gullberg M (1997). Control of microtubule dynamics by oncoprotein 18: dissection of the regulatory role of multisite phosphorylation during mitosis. Mol Cell Biol.

[B14] Howell B, Deacon H, Cassimeris L (1999). Decreasing oncoprotein 18/stathmin levels reduces microtubule catastrophes and increases microtubule polymer in vivo. J Cell Sci.

[B15] Cassimeris L (2002). The oncoprotein 18/stathmin family of microtubule destabilizers. Curr Opin Cell Biol.

[B16] Andersen SSL, Ashford AJ, Tournebize R, Gavet O, A. S, Hyman AA, Karsenti E (1997). Mitotic chromatin regulates phosphorylation of Stathmin/Op18. Nature.

[B17] Budde PP, Kumagai A, Dunphy WG, Heald R (2001). Regulation of Op18 during spindle assembly in Xenopus egg extracts. J Cell Biol.

[B18] Kinoshita K, Habermann B, Hyman AA (2002). XMAP215: a key component of the dynamic microtubule cytoskeleton. Trends Cell Biol.

[B19] Ohkura H, Garcia MA, Toda T (2001). Dis1/TOG universal microtubule adaptors - one MAP for all?. J Cell Sci.

[B20] van Breugel M, Drechsel D, Hyman A (2003). Stu2p, the budding yeast member of the conserved Dis1/XMAP215 family of microtubule-associated proteins is a plus end-binding microtubule destabilizer. J Cell Biol.

[B21] Shirasu-Hiza M, Coughlin P, Mitchison T (2003). Identification of XMAP215 as a microtubule-destabilizing factor in Xenopus egg extract by biochemical purification. J Cell Biol.

[B22] Usui T, Maekawa H, Pereira G, Schiebel E (2003). The XMAP215 homologue Stu2 at yeast spindle pole bodies regulates microtubule dynamics and anchorage. Embo J.

[B23] Kosco KA, Pearson CG, Maddox PS, Wang PJ, Adams IR, Salmon ED, Bloom K, Huffaker TC (2001). Control of microtubule dynamics by Stu2p is essential for spindle orientation and metaphase chromosome alignment in yeast. Mol Biol Cell.

[B24] Walczak CE, Mitchison TJ, Desai A (1996). XKCM1:  A Xenopus kinesin-related protein that regulates microtubule dynamics during mitotic spindle assembly. Cell.

[B25] Desai A, Verma S, Mitchison TJ, Walczak CE (1999). Kin I kinesins are microtubule-destabilizing enzymes. Cell.

[B26] Kline-Smith SL, Walczak CE (2002). The Microtubule-destabilizing Kinesin XKCM1 Regulates Microtubule Dynamic Instability in Cells. Mol Biol Cell.

[B27] Maney T, Wagenbach M, Wordeman L (2001). Molecular dissection of the microtubule depolymerizing activity of mitotic centromere-associated kinesin. Journal of Biological Chemistry.

[B28] Ganem NJ, Compton DA (2004). The KinI kinesin Kif2a is required for bipolar spindle assembly through a functional relationship with MCAK. J Cell Biol.

[B29] Lawrence CJ, Dawe RK, Christie KR, Cleveland DW, Dawson SC, Endow SA, Goldstein LS, Goodson HV, Hirokawa N, Howard J, Malmberg RL, McIntosh JR, Miki H, Mitchison TJ, Okada Y, Reddy AS, Saxton WM, Schliwa M, Scholey JM, Vale RD, Walczak CE, Wordeman L (2004). A standardized kinesin nomenclature. J Cell Biol.

[B30] Wordeman L (2005). Microtubule-depolymerizing kinesins. Curr Opin Cell Biol.

[B31] Moore A, Wordeman L (2004). The mechanism, function and regulation of depolymerizing kinesins during mitosis. Trends Cell Biol.

[B32] Hertzer KM, Ems-McClung SC, Walczak CE (2003). Kin I kinesins: insights into the mechanism of depolymerization. Crit Rev Biochem Mol Biol.

[B33] Wordeman L, Mitchison TJ (1995). Identification and partial characterization of mitotic centromere-associated kinesin, a kinesin-related protein that associates with centromeres during mitosis. J Cell Biol.

[B34] Hunter AW, Caplow M, Coy DL, Hancock WO, Diez S, Wordeman L, Howard J (2003). The Kin I kinesin MCAK is a microtubule depolymerase that forms an ATP-hydrolyzing complex at microtubule ends. Mol Cell.

[B35] Mennella V, Rogers GC, Rogers SL, Buster DW, Vale RD, Sharp DJ (2005). Functionally distinct kinesin-13 family members cooperate to regulate microtubule dynamics during interphase. Nat Cell Biol.

[B36] Moore AT, Rankin KE, von Dassow G, Peris L, Wagenbach M, Ovechkina Y, Andrieux A, Job D, Wordeman L (2005). MCAK associates with the tips of polymerizing microtubules. J Cell Biol.

[B37] Maney T, Hunter AW, Wagenbach M, Wordeman L (1998). Mitotic centromere-associated kinesin is important for anaphase chromosome segregation. J Cell Biol.

[B38] Rogers GC, Rogers SL, Schwimmer TA, Ems-McClung SC, Walczak CE, Vale RD, Scholey JM, Sharp DJ (2004). Two mitotic kinesins cooperate to drive sister chromatid separation during anaphase. Nature.

[B39] Walczak C, Gan EC, Desai A, Mitchison TJ, Kline-Smith SL (2002). The microtubule-destabilizing kinesin, XKCM1 is required for chromosome positioning during spindle assembly. Curr Biol.

[B40] Kline-Smith SL, Khodjakov A, Hergert P, Walczak CE (2004). Depletion of centromeric MCAK leads to chromosome congression and segregation defects due to improper kinetochore attachments. Mol Biol Cell.

[B41] Kapoor TM, Lampson MA, Hergert P, Cameron L, Cimini D, Salmon ED, McEwen BF, Khodjakov A (2006). Chromosomes can congress to the metaphase plate before biorientation. Science.

[B42] Khodjakov A, Copenagle L, Gordon MB, Compton DA, Kapoor TM (2003). Minus-end capture of preformed kinetochore fibers contributes to spindle morphogenesis. J Cell Biol.

[B43] Zhu C, Zhao J, Bibikova M, Leverson JD, Bossy-Wetzel E, Fan JB, Abraham RT, Jiang W (2005). Functional analysis of human microtubule-based motor proteins, the kinesins and dyneins, in mitosis/cytokinesis using RNA interference. Mol Biol Cell.

[B44] Cassimeris L, Morabito J (2004). TOGp, the human homolog of XMAP215/Dis1, is required for centrosome integrity, spindle pole organization, and bipolar spindle assembly. Mol Biol Cell.

[B45] Cimini D, Cameron LA, Salmon ED (2004). Anaphase spindle mechanics prevent mis-segregation of merotelically oriented chromosomes. Curr Biol.

[B46] Waters JC, Mitchison TJ, Rieder CL, Salmon ED (1996). The kinetochore microtubule minus-end disassembly associated with poleward flux produces a force that can do work. Mol Biol Cell.

[B47] Hoyt MA, He L, Loo KK, Saunders WS (1992). Two Saccharomyces cerevisiae kinesin-related gene products required for mitotic spindle assembly. J Cell Biol.

[B48] Kapoor TM, Mayer TU, Coughlin ML, Mitchison TJ (2000). Probing spindle assembly mechanisms with monastrol, a small molecule inhibitor of the mitotic kinesin, Eg5. J Cell Biol.

[B49] Mayer TU, Kapoor TM, Haggarty SJ, King RW, Schreiber SL, Mitchison TJ (1999). Small molecule inhibitor of mitotic spindle bipolarity identified in a phenotype-based screen. Science.

[B50] Sawin KE, LeGuellec K, Philippe M, Mitchison TJ (1992). Mitotic spindle organization by a plus-end directed microtubule motor. Nature.

[B51] Sharp DJ, Yu KR, Sisson JC, Sullivan W, Scholey JM (1999). Antagonistic microtubule-sliding motors position mitotic centrosomes in Drosophila early embryos. Nat Cell Biol.

[B52] Weil D, Garcon L, Harper M, Dumenil D, Dautry F, Kress M (2002). Targeting the kinesin Eg5 to monitor siRNA transfection in mammalian cells. Biotechniques.

[B53] Kittler R, Putz G, Pelletier L, Poser I, Heninger AK, Drechsel D, Fischer S, Konstantinova I, Habermann B, Grabner H, Yaspo ML, Himmelbauer H, Korn B, Neugebauer K, Pisabarro MT, Buchholz F (2004). An endoribonuclease-prepared siRNA screen in human cells identifies genes essential for cell division. Nature.

[B54] Sambrook J, Fritsch E, Maniatis T (1989). Molecular Cloning: A Laboratory Manual.

